# Nontoxic Glucomoringin-Isothiocyanate (GMG-ITC) Rich Soluble Extract Induces Apoptosis and Inhibits Proliferation of Human Prostate Adenocarcinoma Cells (PC-3)

**DOI:** 10.3390/nu10091174

**Published:** 2018-08-27

**Authors:** Mohammed Sani Jaafaru, Nurul Ashikin Abd Karim, Enas Mohamed Eliaser, Peter Maitalata Waziri, Hamidu Ahmed, Mohammed Mustapha Barau, Liliya Kong, Ahmad Faizal Abdull Razis

**Affiliations:** 1 UPM-MAKNA Cancer Research Laboratory, Institute of Bioscience, Universiti Putra Malaysia, 43400 UPM Serdang, Selangor, Malaysia; biojafar@gmail.com (M.S.J.); ck_zimase89@yahoo.com (N.A.A.K.); en_mo2008@yahoo.com (E.M.E.); 2 Department of Biochemistry, Kaduna State University, 2336 Kaduna, Nigeria; petermwaziri@gmail.com; 3 Department of Biology, Faculty of Science, El-Mergib University, El Khums, Libya; 4 Laboratory of Biomolecular Medicine, Institute of Bioscience, Universiti Putra Malaysia, 43400 UPM Serdang, Selangor, Malaysia; boderel@yahoo.com; 5 Department of Biochemistry and Forensic Science, Nigeria Police Academy, Wudil, 3474 Kano, Nigeria; mustahpabar@gmail.com; 6 The Mitomasa Sdn. Bhd., 13-1, Jalan L J 3, Taman Industri Lembah Jaya, 68000 Ampang, Selangor, Malaysia; liliya@live.jp; 7 Laboratory of Food Safety and Food Integrity, Institute of Tropical Agriculture and Food Security, Universiti Putra Malaysia, 43400 UPM Serdang, Selangor, Malaysia; 8 Department of Food Science, Faculty of Food Science and Technology, Universiti Putra Malaysia, 43400 UPM Serdang, Selangor, Malaysia

**Keywords:** acute toxicity, anti-proliferation, apoptosis, cytotoxicity, glucomoringin-isothiocyanate, PC-3

## Abstract

The incidence of prostate cancer malignancy along with other cancer types is increasing worldwide, resulting in high mortality rate due to lack of effective medications. *Moringa oleifera* has been used for the treatment of communicable and non-communicable ailments across tropical countries, yet, little has been documented regarding its effect on prostate cancer. We evaluated the acute toxicity and apoptosis inducing effect of glucomoringin-isothiocyanate rich soluble extracts (GMG-ITC-RSE) from *M. oleifera* in vivo and in vitro, respectively. Glucomoringin was isolated, identified, and characterized using fundamental analytical chemistry tools where Sprague-Dawley (SD) rats, murine fibroblast (3T3), and human prostate adenocarcinoma cells (PC-3) were used for acute toxicity and bioassays experiments. GMG-ITC-RSE did not instigate adverse toxic reactions to the animals even at high doses (2000 mg/kg body weight) and affected none of the vital organs in the rats. The extract exhibited high levels of safety in 3T3 cells, where more than 90% of the cells appeared viable when treated with the extract in a time-dependent manner even at high dose (250 µg/mL). GMG-ITC-RSE significantly triggered morphological aberrations distinctive to apoptosis observed under microscope. These findings obviously revealed the putative safety of GMG-ITC-RSE in vivo and in vitro, in addition to its anti-proliferative effect on PC-3 cells.

## 1. Introduction

Several kinds of vegetative plants are used as folk medicines for the treatment of various disease conditions worldwide [1,2,3,4]. The massive adoption of traditional medicine, especially in Asia, has pointed out the potentials and prospects of plant derived bioactive compounds in the treatment of broad number of disease conditions [5,6]. These compounds are secondary metabolites extracted from specialized vegetative shrubs called medicinal plants situated predominantly in Asia, Africa, South America, and other tropical regions of the world [7,8]. *Moringa oleifera*, popularly known as Drum-stick or Horse-radish tree, is a crucifer vegetable native to the sub-Himalayan region comprising Pakistan, India, Afghanistan, Bangladesh, and some South East Asian tropical countries [1,8,9,10]. The plant has been widely used by ancient communities including the Greeks, Egyptians, Turkish, Africans, Asians, and Romans as a source of medication, in addition to being a dietary supplement source [11,12,13]. The aerial parts (leaves, seeds, and pods) of *M. oleifera* are considered among the major sources of natural nutrients and essential oils [14,15,16], which have been used as a curative to various ailments, thus referred to as the “miracle tree” [17]. Having a high content of nutrient such as vitamins, minerals, proteins, glycosides, and other vital constituents [12,18], coupled with its harmless nature, *M. oleifera* has persuaded the interest of many researchers and has been evaluated for a number of biological activities including, but not limited to, the enhancement of cognitive function, immune-boosting, and wound healing. Others activities include antioxidants, anti-atherosclerotic, anti-diabetic, anti-leukemic, and tumor suppression [1,17,19,20,21].

Generally, crucifier vegetables are rich in glucosinolates, which are sulfur containing glycosylated compounds [22,23,24]. They are hydrolyzed by enzymes called β-thioglucoside-glucohydrolase (myrosinase) in an aqueous environment, to yield biologically more active compounds known as isothiocyanates [22,25]. The hydrolysis products are believed to possess high potency against many communicable and non-communicable disorders [26,27]. Growing number of studies evidenced that consumption of glucosinolate/isothiocyanate rich crucifiers confer protection against various cancer types through the strategic induction of apoptosis to any potential carcinoma [28,29,30,31,32]. Which may be solely attributed to the abundance of naturally occurring glucosinolates and/or isothiocyanates such as allyl isothiocyanate (AITC), phenethyl isothiocyanate (PEITC), benzyl isothiocyanate (BITC), and glucoraphane isothiocyanate (GRFITC) [29]. For instance, AITC has been reported to inhibit the growth of Ehrlich astcites tumors (EATs) through proapoptotic and antiangiogenic pathways [33].

The soluble extract of *M. oleifera* seeds emerged to be rich in glucomoringin (GMG), which is a rare type of glucosinolate commonly found in the plant predominantly the seeds [34]. Furthermore, the hydrolytic product of the glycosylated compound referred to as moringin (MG), glucomoringin-isothiocyanate (GMG-ITC), or 4(α-l-rhamnosyloxy)-benzyl isothiocyanate has shown wide spectrum biological activities including anti-bacterial [35], anti-inflammatory [33], tumor suppressing, and apoptosis inducing activities [36,37] as though the other isothiocyanates [38,39,40].

According to epidemiological reports, human prostate cancer remains among the most highly diagnosed cancer type in European and American male populations [41], and continues to foster significant health care challenges across the world. Despite the advancements in the treatment of cancers, studies have shown that age-related incidence of prostate cancer is on the alarming increase in high, middle, and low income countries [42]. One of the common practices for the treatment of prostate cancer at an advance stage is chemotherapy, which employs the use of chemicals that can trigger apoptosis in tumor cells and flush them out. At present, plant sourced natural products are becoming the preferred approach for the treatment of cancer due to their relative low risk and undesirable effect [41]. However, a limited number of studies have reported on the potential anti-cancer activities of *M. oleifera* aerial parts’ extract, especially the seeds [43]. Therefore, the present study was for the first time designed to investigate the acute oral toxicity and effect of glucomoringin-isothiocyanate rich soluble extract (GMG-ITC-RSE) on cellular proliferation and apoptosis in human prostate cancer cells (PC-3).

## 2. Materials and Methods

### 2.1. Sample Preparation and Characterization

Dried pods of *M. oleifera* were chopped and ground into fine powder particles, and the sample extracted using a modified technique described by Vongsak et al. [44]. Briefly, the dried powder was suspended in cold water in a standard ratio of 1:3 solute to solvent, the mixture was vortex and kept at room temperature for 24 h. The water soluble suspension was removed after centrifugation for 10 min at 5000 rpm. The supernatant was further filtered with Whatman filter paper no 1 and concentrated using a rotary evaporator under reduced pressure. The resulting milky colored extract was freeze dried, packaged, and kept at 4 °C for further analysis.

### 2.2. Nuclear Magnetic Resonance (NMR)

Basically, proton, carbon, and 2-dimensional nuclear magnetic resonance (^1^H, ^13^C, and 2D-NMR) analyses were conducted and the spectra of glucomoringin were verified on a 500 MHz Varian MNR system (Agilent Technologies, Santa Clara, CA, USA). Chemical shifts were standardized relative to deuterium oxide (D_2_O) and expressed in parts per million (ppm).

### 2.3. High Performance Liquid Chromatography (HPLC)

GMG was identified on a Thermo Scientific Dionex Ultimate 3000 UHLC System (Dionex, Sunnyvale, CA, USA) equipped with a C18 column (250 mm × 5.0 mm × 5 μM). An isocratic program of 100% milliQ at 1 mL/min flow rate was performed on GMG-ITC-RSE in multiple ways together with sinigrin (Sigma Aldrich, St. Louis, MO, USA) as the internal standard, and chromatograms were documented.

### 2.4. Liquid Chromatography Mass Spectrometry (LC/MS)

LC/MS analysis was carried out on a Thermo Finningan LCQ Fleet Ion Trap Mass Spectrometer (Thermo Fisher Scientific Inc., Waltham, MA, USA) equipped with a C18 reverse phase column. The compounds were separated using an isocratic program of 100% milliQ similar to that of HPLC. The actual mass of GMG was obtained with ESI detection in the positive mode by injecting 200 μL of sample into the mass spectrometer (6550 iFunnel Q-TOF LC/MS, Agilent Technologies, Santa Clara, CA, USA) at 0.1 mL/min flow rate using a mass spec syringe employing 0.1% formic acid in methanol as the mobile phase.

### 2.5. In Vivo Acute Toxicity

#### 2.5.1. Ethics Statement

The entire animal experiment and sample collections were carried out within the barrier system and a necropsy at Makmal Bioserasi, Center for Research and Instrumentation Management (CRIM), Universiti Kebangsaan Malaysia. The protocols were approved by the University’s Animal Ethics Committee (UKMAEC). The study was conducted in compliance with the appropriate provision of the OECD (2002), Test No. 423: Acute Oral Toxicity-Acute Toxic class method, OECD guidelines for testing of Chemicals Section 4, OECD Publishing Paris.

#### 2.5.2. Animal Grouping and Extract Administration

Inbreed specific pathogen free (SPF) female Sprague-Dawley (SD) rats weighing 249.94 g in the test group, 232.36 g in the continuing test group, and 260.18 g in the control group were obtained from the Animal Breeding Center, Faculty of Science Technology, Universiti Kebangsaan Malaysia. The animals were housed in an appropriate polycarbonate cage with solid bottoms and side with a stainless steel lid as recommended in the Animal Husbandry Guidelines. They were acclimatized for six days under 18.3 °C to 24 °C and 12 h light/dark cycle lightening condition prior to treatment, during which a certified rodent diet (Brastock) and filtered tap water were provided ad libitum. The rats were keep at a one per cage density. All rats neither underwent a previous procedure nor had an abnormal clinical condition prior to study. SD rats were divided randomly into three groups including the test group. A 2000 mg/kg body weight of glucomoringin-isothiocyanate rich soluble extract (GMG-ITC-RSE) in 10 mL/kg body weight pre-filtered water was dosed orally into the test and continuing test groups containing four animals each, whereas control animals were given 10 mL/kg body weight pre-filtered water daily in a similar manner for 14 days.

#### 2.5.3. Body Weight Measurement and Observation of Clinical Signs

During the experiment, the weight changes of the animals, which are an important toxicity index for rats, were measured before grouping, dosing, and sacrifice for statistical analysis. Meanwhile, the clinical signs before and after dosing were carefully monitored in such a way that the animals in the test and control groups were observed for morbidity and mortality at 30 min, hourly, and up to four hours, six hours post dosing, and once daily up to 14 days. Additionally, the animals in the test groups were observed at 30 min, hourly up to four hours, six hours post dosing, and at 24 h. The clinical signs were scored according to the features described in Table 1 and the severity scores were recorded in Table 2.

#### 2.5.4. Necropsy

A complete necropsy was performed on all the animals under study by examining the external features of the carcass and external orifices. The major internal organs were exposed by dissection, and a detailed gross examination of the cranial, thoracic, and abdominal cavities and their contents was performed. The brain, spleen, liver, heart, pancreas, lungs, kidneys, and stomach were appropriately trimmed of any adherent tissue and weighed individually.

### 2.6. In Vitro Study

#### 2.6.1. Cell Culture

The human prostate adenocarcinoma cells (PC-3) and murine fibroblast cells (3T3) used in this study were obtained from the American Type Culture Collection. Cells were grown in RPMI-1640 media (Nacalai, Tesque Inc., Kyota, Japan) supplemented with 10% fetal bovine serum (FBS) and a 1% penicillin (10,000 units/mL)-streptomycin (10,000 μg/mL) mixed solution (stabilized), and were maintained at 37 °C in a 5% CO_2_ and 95% humidified atmospheric air incubator. The PC-3 are stable and immortalized cancer cells, and experiments were conducted when the cells reached 60–80% confluence with not more than 25 passages.

#### 2.6.2. Cytotoxicity and Anti-Proliferation Examination of GMG-ITC Rich Soluble Extract on PC3 and 3T3 Cells

The GMG-ITC rich soluble extract (GMG-ITC-RSE) was tested for cytotoxic and anti-proliferation activities on the PC-3 and 3T3 cell lines employing the MTT assay as a reliable tool for such analysis as described by Devi and Thangam [45]. Briefly, PC-3 and 3T3 cells were seeded separately in 96-wells culture plates at a density of 1 × 10^5^ cells/mL. Following the cells’ attachment after 24 h, 1–100 μg/mL of soluble extract and cisplatin were considered as the selected concentrations for the GMG-ITC-RSE and positive control, respectively, whereas 0.1% DMSO was used as the negative control. The cells were incubated in a 5% CO_2_ and 95% humidified atmospheric air incubator at 37 °C for 24, 48, and 72 h, respectively. The MTT solution (5 mg/mL) was added to the wells thereafter according to the enclosed instructions and incubated for another four hours to allow for the complete formation of the formazan complex by live cells, followed by the replacement of media with DMSO to dissolve the complex formed. All the experiments were conducted in triplicate, where the optical (OD) density was measured on a micro plate reader (Synergy H1, BioTek, Winooski, USA) at 570 nm as described by Satria et al. [46]. The growth inhibition of the test agent is expressed as the IC_50_ value.

#### 2.6.3. Morphological Assessment of GMG-ITC Rich Soluble Extract Treated PC-3 Cells by Inverted Light Microscope

PC-3 cells were seeded in 6-well plates at a density of 2 × 10^5^ cells/mL and incubated overnight for proper attachment. The cells were then exposed to a 2.5 μg/mL GMG-ITC rich soluble extract (GMG-ITC-RSE alongside cisplatin (positive control) for 24, 48, and 72 h, respectively. The treated cells were observed at 20× magnification under an inverted light microscope (Axio Vert A1, Carl-Zeiss-Straße, Oberkochen, Germany), equipped with a camera and image acquisition software (AxioCam MRm, Carl-Zeiss-Straße, Oberkochen, Germany). Multiple images were taken independently.

#### 2.6.4. Morphological Assessment of Apoptotic Cells by Acridine Orange and Propidium Iodide (AO/PI) Double Staining

PC-3 cells were seeded in 6-well plates at a density of 2 × 10^5^ cells/mL, treated, and incubated as described above. Growth media were removed and discarded when the incubation periods elapsed. Cells were then stained according to the enclosed protocol using 10 μL from the mixture of 10 μg/mL AO and PI each, respectively. The stained cells were assessed using an inverted fluorescence microscope (Axio Vert A1, Carl-Zeiss-Straße, Oberkochen, Germany) equipped with a digital imaging system (AxioCam MRm, Carl-Zeiss-Straße, Oberkochen, Germany) and multiple images were also taken independently.

### 2.7. Statistical Analysis

SPSS statistical software version 22.0 (SPP Inc., Chicago, IL, USA) was used for the analysis and the data presented were taken in triplicate independently. Comparison of means in the acute toxicity study was performed using one way ANOVA employing the Post Hoc feature, whereas the data from the cytotoxic activities of the GMG-ITC rich soluble extract, cisplatin, and 0.1% DMSO on PC-3 cells was performed using the student *t*-test and *p* < 0.05 was considered statistically significant.

## 3. Results

### 3.1. Chemical Profiling of GMG-ITC Rich Soluble Extract (GMG-ITC-RSE)

**Figure 1:**
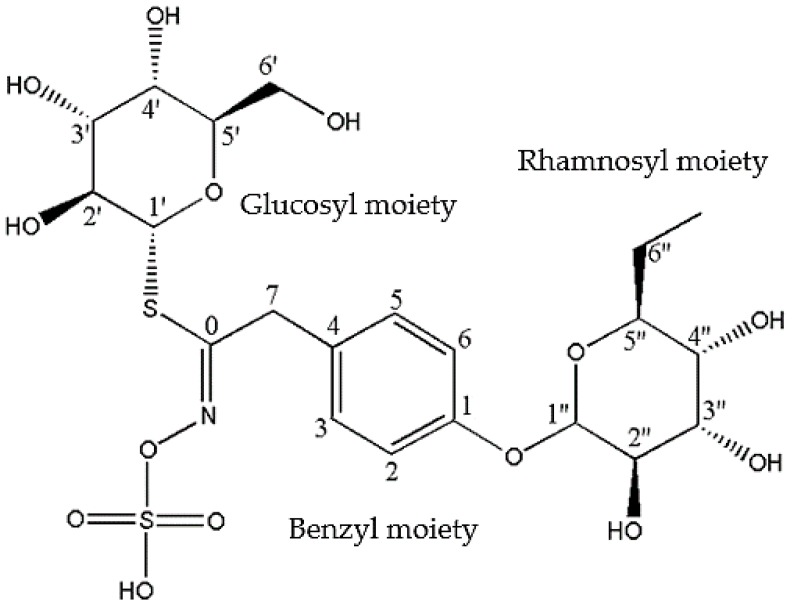
Intact structure of glucomoringin comprising glucosyl, benzyl, and rhamnosyl moieties. Adapted from Jaafaru et al. [24].

**Figure 2:**
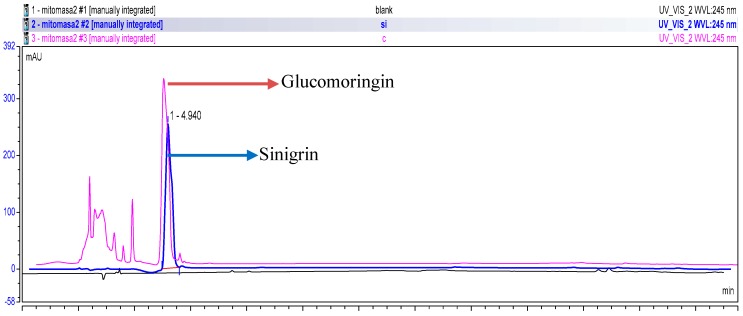
High performance liquid chromatography of the GMG-ITC rich soluble extract rich in at 245 nm. The blue line represents the elution in progress of sinigrin (standard), the black line is for the blank, while the pink line indicates the elution progress of the soluble extracts. Retention time (RT) for the targeted compounds was between 5.5–6.0 min.

**Figure 3:**
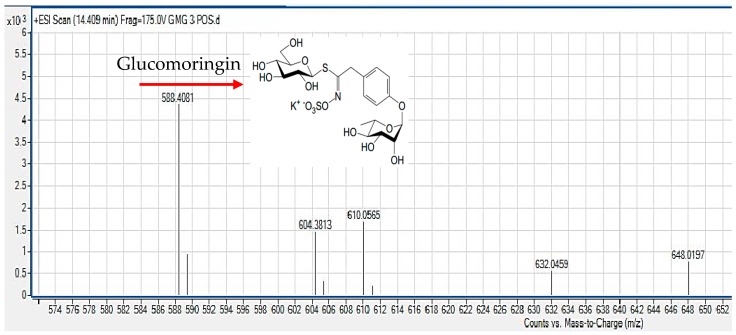
Liquid chromatography mass spectrometry (LC/MS) analysis of GMG-ITC-RSE showing the molecular ion peak at *m*/*z* 588 (positive mode), corresponding to the molecular formula of GMG reported by de Graaf et al. [48].

NMR analysis was conducted on the GMG-ITC-RSE, and the ^1^H-NMR and ^13^C-NMR spectra for GMG were observed and the chemical shift data were recorded and presented in Table 3. Numbering of carbon atoms in the table was done according to the individual moieties that made up the structure shown in Figure 1. The conformation of rhamnosyl and that of glucose moieties were confirmed considering the J-couplings of hydrogens attached to position 1 to 5 of the moieties. The ^1^H, ^13^C and 2D: heteronuclea single quantum coherence (HSQC) and correlation spectroscopy (COSY) NMR analyses provided clear peaks that differentiated the rhamnose to glucose moieties in addition to other peaks typical of aromatic rings, particularly benzene, which signifies the presence of glucosinolates containing sugar moieties. HPLC analysis of GMG-ITC-RSE revealed a peak around 4.940 min (retention time) at 245 nm, similar to that of sinigrin (4.940 min), the internal standard (Figure 2), as reported by [47], which indicated the presence of glucosinolates in the extract. Identification of the *M. oleifera* derived glucosinolate under investigation was further corroborated by mass spectrometric examination where LC/MS ESI analyses in the positive mode revealed an *m*/*z* 588 (M + H^+^) (Figure 3), which corresponded to the mass of intact GMG (Figure 1) reported by [48]. The combination of NMR, HPLC, and LC/MS analytical data therefore confirmed the presence of 4-(a-rhamnosyloxy) benzyl glucosinolate, known as GMG, in the extract.

### 3.2. Survival, Observations, and Body Weight of Animal after GMG-ITC Rich Soluble Extract Dosage

**Figure 4:**
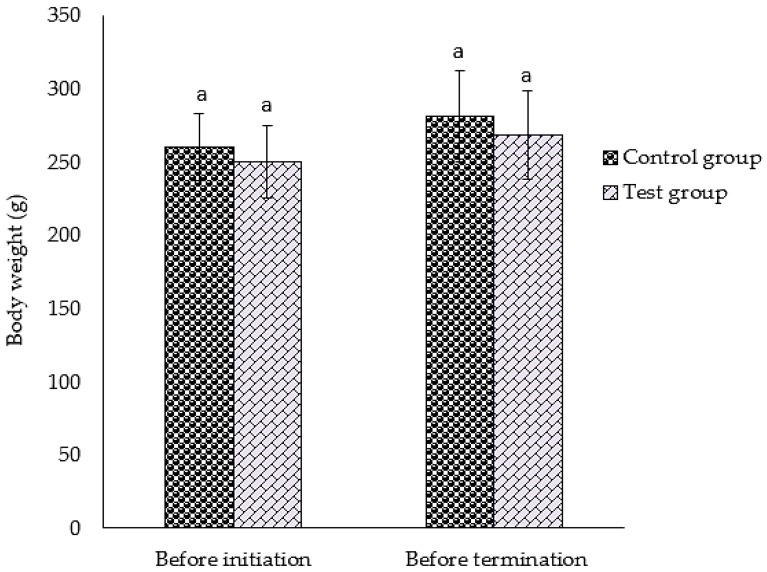
Body weight of animals in the control group and test group measured before the initiation and termination of the study. Results are presented as mean ± SD body weight, while *p* < 0.05 is considered statistically significant. Means superscripted with the same letter (a) are not different (*p* > 0.05) statistically unless stated otherwise.

After high levels of extract dosage, the survival of animals under investigation and clinical observations were made where all of the animals in the test group, continuing test group, and control group survived the course of the study period with no evidence of either clinical or toxic manifestation as shown in Table 1 and Table 2 in the previous section. However, there was no sign of weight loss when the weight of animals in the test and control groups was measured at two intervals (before treatment and termination of the experiment) at the study period as reported in Figure 4.

### 3.3. Effect of GMG-ITC Rich Soluble Extract on Animal Organs’ Weight

**Figure 5:**
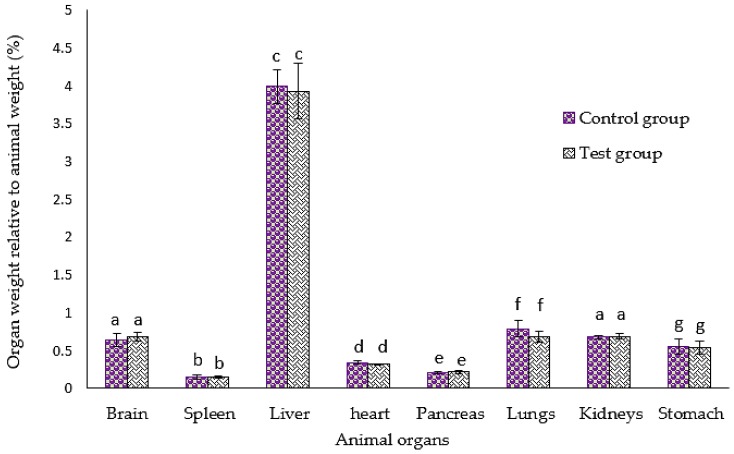
Comparison of organ weight relative to animal body weight in the test and control groups measured during the necropsy experiment. Results are presented as mean ± SD in percentage. While *p* < 0.05 is considered significant, similar letters on the means in every cluster indicated no significant (*p* > 0.05) difference between the two means unless stated otherwise.

Result of the gross examination of all the animals in the test group and control group revealed no remarkable abnormalities in the investigated organs, which included the brain, kidney lungs, liver, stomach, spleen, heart, and pancreas when subjected to necropsy. Moreover, no significant (*p* > 0.05) difference in the mean percentage of organ weight to animal body weight between the test group and the control group was observed as shown in Figure 5. The safety of *M. oleifera* was deeply explained by the accumulated number of studies reported by Stohs and Hartman in [14].

### 3.4. Cytotoxicity and Anti-Proliferation Evaluation of GMG-ITC Rich Soluble Extract

**Figure 6:**
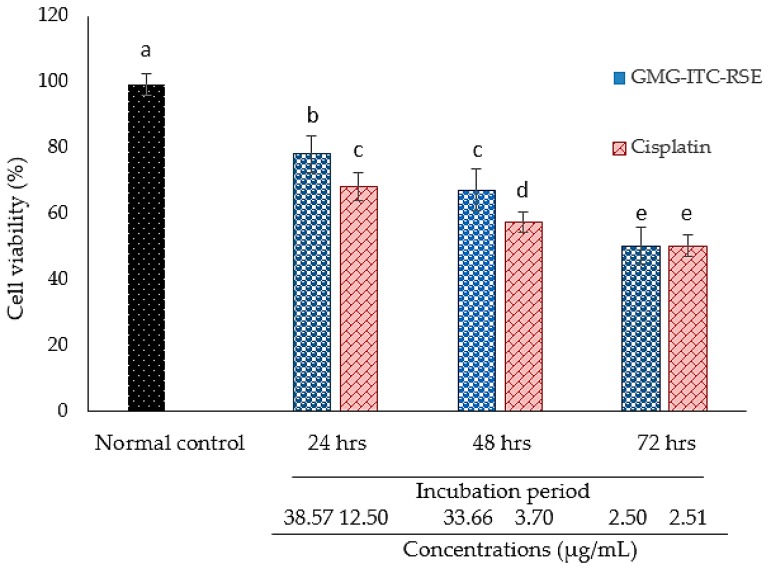
Cytotoxicity effect of the GMG-ITC rich soluble extract (GMG-ITC-RSE) on the PC-3 cell lines. The cells were treated with GMG-ITC-RSE and cisplatin for 24, 48, and 72 h, respectively. Results are presented as mean ± SD percentage viability. While *p* < 0.05 is considered statistically significant, similar letters on the means in every cluster indicated no statistically significant (*p* < 0.05) difference between the two means, unless stated otherwise.

**Figure 7:**
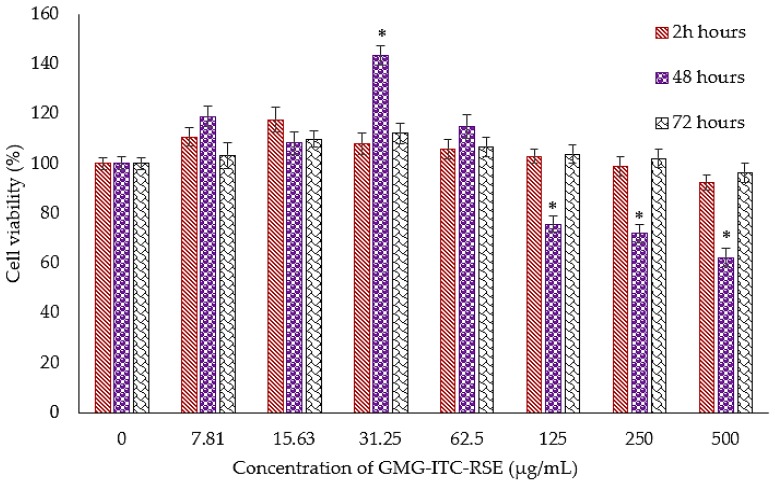
Cytotoxicity effect of the GMG-ITC rich soluble extract (GMG-ITC-RSE) on the murine fibroblast (noncancerous) cell lines (3T3). The cells were treated with GMG-ITC-RSE for 24, 48, and 72 h at varying concentrations (0 to 500 µg/mL). Results are presented as the means of percentage viability, where the means with asterisks are significantly (*p* < 0.05) different from the normal control unless stated otherwise. Meanwhile *p* < 0.05 was considered statistically significant in the analysis.

The MTT result demonstrated an impressive cytotoxic activity of GMG-ITC rich soluble extract (GMG-ITC-RSE) against the PC-3 cell lines, where the cells were exposed to various concentrations in μg/mL of the extract and cisplatin periodically in a time dependent manner. Results showed that the IC_50_ of GMG-ITC-RSE appeared to be 2.5 μg/mL after 72 h of incubation and was similar to that of cisplatin obtained at the same period shown in Figure 6. However, Figure 7 displays the percentage of the cell’s viability after exposure to 0 to 500 µg/mL GMG-ITC-RSE for 24, 48, and 72 h. The data indicated that GMG-ITC-RSE did not have an IC_50_ value on the 3T3 cell lines due to the inability of the extract to inhibit the growth of 50% of the cells. Moreover, the growth of 37.78% cells was inhibited when exposed to 500 μg/mL GMG-ITC-RSE for 48 h, which was contrary to 72 h (<4%).

### 3.5. Morphological Assessment of PC3 Cells by Inverted Light Microscope

**Figure 8:**
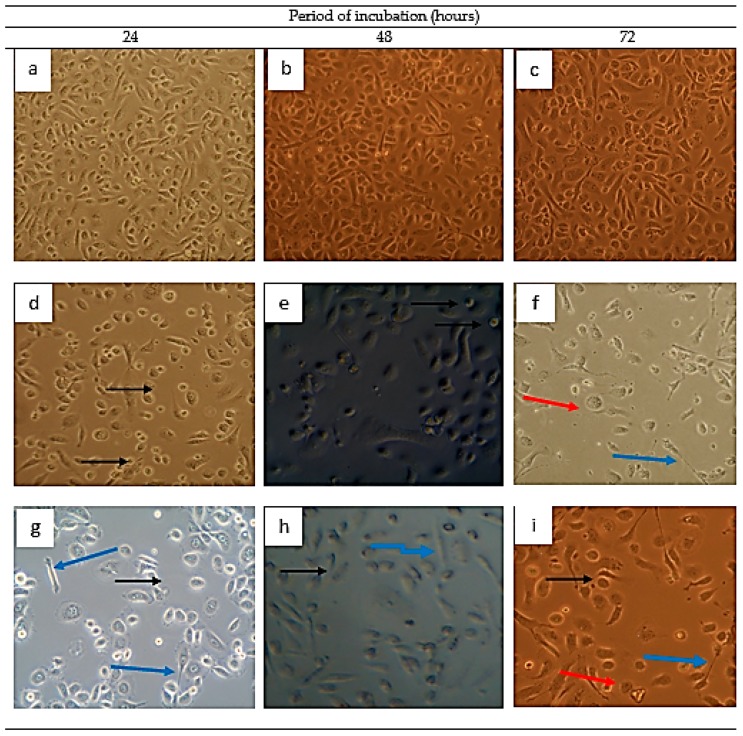
Micrographs of apoptosis assessment on the PC-3 cell lines using a phase contrast microscope. (**a**–**c**) represent the untreated control cells after 24, 48, and 72 h. (**d**–**f**) show cells treated with 2.51 µg/mL cisplatin (standard drug) for the respective 24, 48, and 72 h. Whereas, (**g**–**i**) of the same figure showcase treatment with 2.5 µg/mL GMG-ITC-RSE on PC-3 for 24, 48, and 72 h. Black arrow = condensed chromatin in nuclei with occasional enucleation, blue arrow = elongated lamellipodia, and red arrow = cells are detaching from substrate, 20× magnification.

The PC-3 cell lines were observed under phase contrast using an inverted light microscope for the assessment of plasma membrane integrity, where morphological changes resulting from the GMG-ITC rich soluble extract (GMG-ITC-RSE) and cisplatin treatment in a time dependent manner were recorded. Figure 8g–i represent the 2.5 μg/mL GMG-ITC-RSE treated cells for 24, 48, and 72 h, where the integrity of plasma membranes declined, leading to obvious morphological alterations in the cells when compared to the normal morphology observed in untreated cells (control) displayed in Figure 8a–c. Condensed chromatin with occasional enucleated (pointed in black arrow), elongated lamelliopodia (blue arrow), and cellular detachment from the substrate (red arrow) were manifested in both the GMG-ITC-RSE (Figure 8g–i) and cisplatin (in Figure 8d–f). A drastic decrease in the number of live cells at 72 h post treatment and cellular-to-substrate detachment observed in the test and positive control treated groups indicated a clear similarity of the mode of action between the commercially available drug (cisplatin) and the extract under investigation (GMG-ITC-RSE).

### 3.6. Morphological Assessment of Apoptotic Cells by AO/PI Double Staining

**Figure 9:**
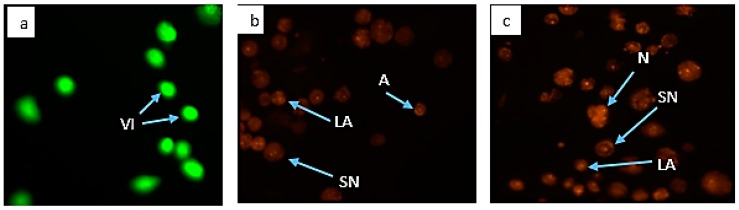
Fluorescence micrographs of apoptosis assessment using acridine orange and propidium iodide staining dyes on the PC 3 cell lines. (**a**) Untreated cells, (**b**) Cells treated with 2.5 μg/mL (IC_50_) GMG-ITC-RSE for 72 h, and (**c**) 72 h post-incubated cells with 2.5 μg/mL (IC_50_) cisplatin. Where A, stands for apoptosis; LA, late apoptosis; N, necrosis; SN secondary necrosis; and VI, viable cells. The images were taken at 20× magnification.

AO/PI double staining of PC-3 cell lines clearly differentiated viable, necrotic, and apoptotic cells in the present study. Untreated (control) cells exhibited round greenish nuclei in Figure 9a, indicating healthy and live cells. Secondary necrosis and apoptosis were observed in both 2.5 μg/mL GMG-ITC-RSE (Figure 9b) and 2.5 μg/mL cisplatin (Figure 9c) treated cells for 72 h. The displayed images of AO/PI double stained treated cells revealed the capacity of GMG-ITC-RSE to decrease the proliferative potentials of PC-3 cell lines, thereby projecting the ability of the lead compounds to treat and prevent carcinogenesis, particularly human prostate cancer.

## 4. Discussion

The growing number of studies on pharmacology and epidemiology has exposed the potential health enhancing properties of plant derived natural compounds [3,8,24,49]. Various phytochemicals, particularly glucosinolates and their hydrolytic products (isothiocyanates), have been promising metabolites demonstrating a drastic decrease in the susceptibility to carcinogenesis [39,40]. Glucosinolates and isothiocyanates including, but not limited to, glucoraphanin, benzyl isothiocyanate, phenethyl isothiocyanate, allyl isothiocyanate, and sulforaphane have been used as strong chemotherapeutic and preventive measures against various types of carcinoma [50,51,52,53]. These sorts of phytochemicals and other potent bioactive compounds are believed to have a modulatory effect on the cancer hallmarks such as angiogenesis, metastasis, and tumor cell proliferation through the induction of a cascade of reactions that trigger intracellular ROS generation leading to the downregulation of various antioxidant genes with consequent cell cycle arrest and eventually death via apoptosis [54,55]. Prostate cancer is a complex disease that affects mostly aged individuals, particularly males, and is associated with an increase in the rate of mortality worldwide [56]. The current treatment of human prostate cancer is shouldered on radiation and chemotherapy after surgery [57,58]. Despite several ways of managing the disease, recurrent tumors have always been a concern to cancer patients, distressing a large percentage of individuals undergoing treatment [58]. Therefore, alternative approaches that employ safer plant-derived natural compounds have become a better option [59]. Thus, using a *M. oleifera* extract rich in glucomoringin and or its hydrolytic product, which have demonstrated promising biological activities against human prostate carcinoma, appears to be a potential substitute to the existing practice believed to be less effective.

Numerous in vivo and in vitro studies have reported on the various anti-tumor activities of extracts of aerial parts of *M. oleifera* [43,60,61,62], which have been proven to be non-toxic as reported by Stohs and Hartman [14]. However, the anti-cancer properties of the glucomoringin rich soluble extract against human prostate carcinoma remained ambiguous. As such, this study reported on the toxicity and antitumor effects of glucomoringin-isothiocyanate rich soluble extracts (GMG-ITC-RSE) by employing inbreed specific pathogen free (SPF) female Sprague-Dawley (SD) rats, murine fibroblast cell lines (3T3), and human prostate adenocarcinoma cell lines (PC-3) as apparently normal and disease models, respectively. After examining the potential toxicological effects of the aqueous GMG-ITC-RSE of *M. oleifera* seeds on SD rats, where oral doses of up to 2000 mg/kg body weight were administered to the rats in the test group and continuing test group for 14 days. We noticed that there were neither observed overt adverse reactions at this dose nor was any organ enlargement found when the major organs underwent necropsy. Thus, our findings were in line with what was reported by Stohs and Hartman [14], where they considered not only the seeds of *M. oleifera*, but all aerial parts of the plant as nontoxic and safe enough to move on with clinical studies after the exploration in all respects. The authors gathered that numerous studies on human and animal models have evaluated the general safety of *M. oleifera* aerial parts, particularly the leaves and seeds, thus demonstrating a high level of safety. Their reports explained how safe the leaves of the plant were when administered in humans as the result revealed no adverse effect on doses of up to 50 g [63]. Similarly, oral administration of up to 8 g/day of the leaf powder for 40 days recorded no toxic effects [64]. Although *M. oleifera* extracts of any solvents has yet to be tested in humans, in-depth clues on the safety of extracts of different solvents were obtained when the extracts were administered in various animal models. Approximately, 300 mg per kg body weight of *M. oleifera* leaves and seeds in aqueous extracts, which was equivalent to 3.9 g per 80 kg human BW, was administered in rats [43]. The findings showed that extracts of the *M. oleifera* aerial parts could be very safe in addition to their potential physiological and pharmacological effects.

We realized that the soluble extract offered an anticancer effect through the induction of morphological alterations and apoptosis in a time dependent manner. After treatment of the PC-3 cells with GMG-ITC-RSE and incubation for 24, 48, and 72 h, we observed noticeable morphological changes at 72 h of incubation. We also observed a deeply stained nuclei and condensed chromatin, which is an indication of the processes that dying cells undergo. The acridine orange (AO) and propidium iodide (PI) double stain confirmed the chromatin condensation and other features peculiar to particular stages of processes for cell death through apoptosis as seen clearly in Figure 8. A similar trend of activity was reported when pancreatic cancer cell lines [65] and human brain glioblastoma multi form cells [66] were incubated with BITC, and glioma cells with PEITC [53], where the PEITC was found to significantly potentiate the induction of cytotoxicity and apoptosis in glioma cells through the agonistic effect of the tumor necrosis factor-related apoptosis-induced ligand (TRAIL).

Although our study did not establish the specific pathway(s) for the signal transduction in the apoptosis process due to some limitations, the accumulated number of findings suggested certain mechanisms through which *M. oleifera* extracts influence cellular proliferation and the number of genes/signaling proteins involved [67,68]. Meanwhile, according to Jung [43], the soluble extract of *M. oleifera* remarkably upregulated the expression of cleaved caspase 3 with the consequent downregulation of caspase 3 when treated with lung and other cancer cell lines at different doses. Furthermore, the extract was reported to have triggered apoptosis through the activation of phosphor-extracellular signal related kinase (p-ERK) and phosphor-c-Jun N-terminal kinase (pJNK) in human melanoma cancer cells (A2058) [69]. Similarly, an extract from *M. oleifera* seeds caused an increase in the phosphorylation level of ERK1/2 and p38 MAPK, which led to apoptosis in RMCCA1 [70]. The authors suggested that the activities of pro- and anti-apoptotic markers could be the benchmark for the apoptotic nature of the extract.

However, studies have shown that the major worrying factor in the drug development process and discovery is the water insolubility of potential anti-cancer agents due to the presence of bulky hydrophobic moieties in their structures [71,72], which reflect difficulties in biotransformation with eventual therapeutic challenges [73]. The more soluble the compounds are, the better their efficacy in cancer treatment and prevention will be. As such, the high polarity of glucosinolate hydrolysis products qualify it to dissolve easily in water, which gives it immense anti-tumor activity [74] in the presence of other related compounds as was mentioned earlier.

## 5. Conclusions

We demonstrated the in vitro and in vivo acute toxicity coupled with in vitro anti-tumor activity of a nontoxic glucomoringin-isothiocyanate rich soluble extract from *M. oleifera* seeds on human prostate adenocarcinoma through proliferative inhibition and apoptotic induction in PC-3 cells. The findings suggested that the soluble extract of *M. oleifera* seeds possessed a high level of safety and promising physiological and pharmacological activities. The plant could be a potential source of a solution to the challenges facing the current treatment of prostate cancer and perhaps other types of cancer conditions. The highly noticeable antitumor activity of the soluble extract could be attributed to the synergistic effect of GMG-ITC with other potent compounds in the extract that may enhance its activities. However, in-depth investigation at the cellular and molecular level are essential to address the points brought up in the discussion section.

## Figures and Tables

**Table 1:** Gross outlined features for clinical observations.

Reaction	Score	Severity
Fur and skin changes	A	0
Eyes changes	B	0
Mucous membrane	C	0
Pale	D	0
Congested	a	0
Motor activity	E	0
Increased motor activity-speed of movement increases	a	0
Decreased motor activity—lethargy, does not respond to external stimuli	b	0
Tremor (continuous repetitive twitching of skeletal muscles, usually palpable and visible)	F	0
Convulsion (Involuntary contraction of the voluntary muscle)	G	0
Tonic convulsion—sustained spasm with head arched backward	a	0
Clonic convulsion—short choppy spasm with head arched toward stomach	b	0
Mixed convulsions—combination of tonic and clonic convulsions	c	0
Walking backwards and/or ataxia, inability to coordinate bodily movements (gross wobbling)	H	0
Diarrhea	I	0
Death—self-explanatory	J	0
Others	K	0

Where A–K and a–c refer to the respective main and sub studied clinical features. The digits in column 3 signify the scores for severity of the condition (if any) as defined in Table 2 below, with 0 being a normal/unchanged condition.

**Table 2:** Severity score of observed changes.

Severity	Score
Normal/No changes	0
Mild	1
Moderate	2
Severe	3

**Table 3:** ^1^H-NMR and ^13^C-NMR spectroscopic data for glucomoringin in soluble extract of *M. oleifera* seeds.

Individual Moieties	NMR Chemical Shifts de Graaf et al. [45]		NMR Chemical Shifts (Present Study)	
	δC	δH (J in Hz)	δC	δH (J in Hz)
**Glucose Moiety**				
1′	80.9	4.57, d, (9.0)	80.4	4.57, d
2′	71.9	3.17–3.30, m	71.8	3.17–3.36, m
3′	77.0	3.17–3.30, m	76.9	3.17–3.36, m
4′	68.8	3.17–3.30, m	68.6	3.17–3.36, m
5′	79.7	3.09, m	79.7	3.09, m
6′	60.2	3.50, m	60.2	3.49, m
**Benzyl Moiety**				
1	154.4	—	154.5	—
2	117.4	7.00, d, (8.7)	117.5	7.00, d (8.7)
3	129.3	7.18, d, (8.7)	129.4	7.21, d, (8.7)
4	130.4	—	130.7	—
5	129.3	7.18, d, (8.7)	129.4	7.21, d (8.7)
6	117.4	7.00, d, (8.7)	117.5	7.00, d, (8.7)
7	37.2	3.86, d, (17.0)	37.4	3.86, d, (17.0)
**Others**				
0	154.6		154.5	
**Rhamnose Moiety**				
1″	98.1	5.41, d, (1.7)	98.0	5.41, d, (1.7)
2″	69.9	4.03, d, (1.7–3.5)	69.6	4.02, d (1.7–3.5)
3″	70.0	3.86, d, (3.5, 7.0)	69.9	3.86, d, (3.5–7.0)
4″	70.2	3.38, t, (9.7)	70.4	3.36, t, (9.7)
5″	69.3	3.67, m	69.3	3.65, m
6″	16.6	1.09, d, (6.3)	16.6	1.08, d (63)

Where d stands for double, m for multiple, and t for triple peaks, respectively.
